# Deciphering the role of soil temperature dynamics in modulating cotton (*Gossypium hirsutum L.*) growth, yield, and resource efficiency under variable sowing dates

**DOI:** 10.1186/s12870-026-08648-x

**Published:** 2026-06-24

**Authors:** Hamad Khan, Madjebi Collela Be, Zeeshan Khan, Yingchun Han, Beifang Yang, Yaping Lei, Xiaoyu Zhi, Shiwu Xiong, Shilong Shang, Yunzhen Ma, Yahui Jiao, Tao Lin, Yabing Li

**Affiliations:** 1 Zhengzhou Research Base, State Key Laboratory of Cotton Bio-breeding and Integrated Utilization, School of Agriculture and Bio-Manufacturing, Zhengzhou University, Zhengzhou, China; 2 State Key Laboratory of Cotton Bio-breeding and Integrated Utilization, Institute of Cotton Research, Chinese Academy of Agricultural Sciences, Anyang, 455000 Henan China; 3 Atta-ur-Rahman School of Applied Biosciences (ASAB), National University of Sciences and Technology, Islamabad, 44000 Pakistan; 4 Institute of Cash Crops, Xinjiang Academy of Agricultural Sciences, Urumqi, China

**Keywords:** Sowing date optimization, Cotton yield, Resource use efficiency, Climate change, Thermal accumulation, Biomass accumulation

## Abstract

**Background:**

Sowing period has a considerable effect on cotton productivity by regulating the use of thermal resources, but its effect on various soils depth layers has not been properly measured. The current study investigated the impacts of date sowing on cotton yield, cumulative growing degree days (GDD) and soil temperatures using a multi-depth monitoring system.

**Methods:**

A field study (2022–2024) was chosen to take place in Anyang, Henan Province, China, on the North China Plain, where the climate is that of a temperate continental monsoon. In a randomized block trial, the cotton cultivar CCRI 134 was sown on six different dates; 12 April (S1), 12 May (S6). Mean daily temperature and rainfall during the growing season were 21.9 °C and 656.6 mm in 2022, 23.66 °C and 793.5 mm in 2023, and 23.2 °C and 315.9 mm in 2024.

**Results:**

Seed cotton yield varied significantly with sowing date, but the optimum differed by year: S3 yielded 3025.9 kg ha⁻¹ in 2022, S5 yielded 3398.5 kg ha⁻¹ in 2023, and S2 yielded 2561.7 kg ha⁻¹ in 2024, compared with 2118.4, 2815.5, and 1506.8 kg ha⁻¹ under S6, respectively. Earlier sowing reduced thermal accumulation under cooler conditions. Soil thermal responses were depth-dependent, with stronger variation in the 0–30 and 30–70 cm layers, while 70–110 cm remained more stable.

**Conclusions:**

Multi-depth soil temperature monitoring showed that very late sowing should be avoided, and sowing between 18 April and 6 May is the most reliable window for balancing soil heat accumulation and yield.

## Introduction

Optimizing the date of sowing is a critical agricultural field management practice that significantly affects both crop productivity and resource allocation. It helps crops grow more efficiently and utilize resources more effectively [1]. The date of sowing directly impacts how crops grow and develop, which in turn influences the amount they produce and their efficiency in resource use [2]. For example, cotton *(Gossypium hirsutum L.)* is significantly impacted by the growing environment depending on the sowing date. Early cotton seeding may expose the crops to greater temperatures and more humidity during flowering and boll development, thereby improving yield [1]. In contrast, late-seeded cotton is more likely to encounter lower temperatures during the late flowering and boll development stages, which could result in lower yields [3]. Moreover, the total number of leaves and the leaf area are two additional crop growth parameters that are affected by the timing of sowing. Late-seeded cotton has fewer leaves per plant than early and middle-seeded cotton [4]. Reportedly, the timing of cotton sowing has a direct impact on biomass accumulation, with a steady decrease observed as planting is postponed [5]. Timely planting of cotton plants enables them to maximize the use of available light, temperature, and water resources, which improves overall establishment and production. For example, it has been demonstrated that early sowing increases growing degree days (GDDs), which maximizes GDDs and reduces cool hours during boll formation, leading to more yields and better fiber quality [6]. On the other hand, late sowing may result in a longer development stage and possible yield decreases by lengthening the time between flowering and boll opening [7]. This tendency is most pronounced in early-maturing types, while medium to late-maturing cultivars require more specific planting dates [8]. However, early sowing can have a detrimental effect on seedling growth as it exposes the crop to lower temperatures and increased insect pressure [9]. Additionally, because late seeding hinders the plant’s capacity to support reproductive growth efficiently, it has been used to delay cotton maturity and lower output because of inadequate carbohydrate synthesis [10]. These findings underscore the importance of adjusting sowing dates to enhance cotton yield and quality.

A major crop worldwide, cotton *(Gossypium hirsutum L.)*, is the primary source of fiber used in the textile industry. Climate variables, including temperature, light, and water availability, have a significant impact on their growth and yield. Sustainable cotton production relies on maximizing the efficient use of these resources, particularly in light of climate change and increasing water constraints [11]. Temperature is an important factor in cotton growth. Studies have reported that the ideal temperature range for metabolic activity is 23–32 °C, with maximal photosynthesis occurring at 28 °C. Temperatures above 35 °C can greatly hinder seed emergence and overall development [12]. Growing degree-days (GDD) are frequently used as an indicator for assessing crop growth because of the close relationship between temperature and plant development. Sowing date variations have a considerable impact on GDD buildup, which affects the crop’s phenological development and heat usage efficiency [13]. GDD changes throughout distinct growth phases and may be used to precisely estimate the time of certain development stages of a crop at a given site [14]. The heat use efficiency index may also be used to anticipate the occurrence of phenological stages [15]. Photosynthetically active radiation (PAR) is another vital element that influences cotton biomass accumulation. Recent studies have emphasized the significant impact of sowing date on cotton’s interception of PAR, which influences yield formation [16]. For example, delaying sowing might shorten the growth season, resulting in lower PAR accumulation and consequent yield losses. In contrast, adjusting the sowing date can improve PAR interception, hence enhancing photosynthetic efficiency and total cotton yield [17]. Soil water content (SWC) is directly related to the development phases of cotton. Adequate hydration during the blooming and boll development stages is critical for increasing production. Research has shown that maintaining sufficient SWC levels may significantly increase cotton biomass and seed cotton yield [18]. Soil and air temperature, as well as soil moisture consumption, can all influence the development of cotton reproductive organs, stem and leaf growth, and growth of belowground organ growth [19]. The soil temperature in the root zone should be within the optimal range in order to maintain normal root activities and crop growth. Recent research indicated that the optimal soil temperature at which cotton roots develop is about 28–32 °C, and a lower temperature of less than 10 °C or higher temperature of 38 °C may suppress the metabolic activities of roots and slow down the growth of the plant [20–22]. Variation in soil temperature at different depths can also impact root growth and nutrient uptake, impacting overall plant health and production potential. According to research, maintaining optimal soil temperatures throughout the growing season enhances cotton’s ability to utilize water and nutrients efficiently, leading to increased yield [18]. These parameters are closely associated with the metabolism and physiological activity of the cotton crop, as well as its own metabolism and physiological activity [23].

In the context of increased climate change, the frequency and intensity of extreme weather events, such as extreme temperatures, droughts, and erratic rainfall, render circumstances less conducive to good agricultural development [24]. These changes pose substantial obstacles to cotton production, especially during critical development periods. According to research, high temperatures, particularly those exceeding 32 °C, can have a detrimental effect on cotton crops, especially during the reproductive stage [25]. Furthermore, increasing temperatures hasten cotton’s phenological development, shortening its growth cycle and perhaps diminishing yield [26]. To mitigate the negative consequences of climate change, optimal planting dates and effective resource management methods are necessary. Adjusting sowing dates can help coordinate cotton growth with favorable weather conditions, thereby improving resource use efficiency [2].

Recent studies have shown that the alleged benefits of early or late sowing are not inherently contradictory, but they are mostly dependent on climatic peculiarities of the region, the general characteristics of the cultivar, and seasonal thermal conditions. Indicatively, multi-year field experiments have shown that best cotton yield may be achieved with various planting in different years because of differences in thermal resources, rainfall patterns, and soil heat dynamics vary between seasons [16]. Similarly, several recent studies state that late sowing can reduce productivity by shortening the effective vegetative time and exposing the reproductive stages to adverse late-season environments [27], and early sowing can also improve yield when the soil temperatures are high enough to allow seedlings to grow and roots to develop in a short time [28]. These findings make it clear that the results of sowing-date are best viewed within the framework of existing soil-atmospheric thermal regime, rather than assuming a universally optimal planting time. Root-zone temperature has a dire effect on crop performance as it directly controls root development, osmotic uptake of water, and nutrient assimilation. The root activity can be altered in response to variations in soil temperature within the root zone, which consequently alters the biomass accumulation and yield development. Previous studies have also shown that inappropriate temperatures of the soil suppress the growth of roots and limit the absorption of water and nutrients, thus lowering crop yields [29, 30]. Besides, the constant temperature in deep soil layers contributes to the maintenance of root functionality in the later stages of growth and could elevate crops performance in a range of environmental conditions [31].

Air temperature primarily modulates above‑ground physiological processes, whereas soil temperature exerts direct control over root activity, water uptake, and nutrient absorption. Since the temperature of the soil is relatively steady and, in many cases, more closely related to the establishment of crops and root growth, soils have a higher thermal inertia than the atmosphere [22]. Comprehensive research on the combined effects of light, heat, and water across various soil profiles and developmental stages (0–30 cm, 30–70 cm, and 70–110 cm), as well as how yield, biomass, and resource use efficiency react to the best dates for sowing under various climate conditions, is still lacking, though. Below the upper and middle soil layers, there are subsoil layers more than 70 cm deep and these layers act as relatively stable stores of thermal energy and moisture and therefore counter the transitory surface-condition fluctuations. The existence of thermal heterogeneity decreases in depth because of higher thermal inertia and consequently creates more stable thermal regimes that oscillate root growth, assimilation of nutrients, and crop production in response to varying environmental conditions [32]. Moreover, deep-rooted species can utilize water that is stored in subsoil layers (60–100 cm) between phenological stages, an asset that is also valued during advanced phenological stages or during water deficit, thus increasing yield consistency and efficiently utilizing resources [33, 34]. Recent field research has shown that root biomass and activity are often observed to extend to depths of up to one metre and that the physicochemical properties at these depths often have significant impact on crop performance, making it important to include deeper horizons in agronomic research investigations [35]. It is therefore necessary to incorporate the 70–110 cm layer of soil in a study, to gain a more holistic understanding of soil thermal processes and how they affect cotton to grow, form yields, and develop their allocation at different sowing times.

The increased crop productivity that must be achieved in the face of a changing climate requires agronomic changes that would strengthen climatic resilience particularly through the optimal exploitation of the soil thermal and moisture reserve. The time synchronization of sowing with soil temperature dynamics is one of the key adaptation strategies that would help to address the negative effect of temperature change on physiological growth and yield of crops [36–38]. This means that systematic evaluations of the temperature contexts in the soil under stratified depths in respect to the various times of sowing can be used to develop climate resistant cotton production systems. The effect of temperature change and the timing of sowing on crop growth and development have been reported in previous studies, but most studies have focused on atmospheric temperature or surface layers of soil, whereas few studies have reported outcomes of soil temperature dynamics at deeper depths and relating to crop development, yield formation and resource use efficiency with respect to different sowing dates. The role of subsurface thermal conditions (> 70 cm ) in shaping crop performance in varying environmental conditions is of special concern and is currently not adequately explained. Therefore, it is necessary to have a more thorough evaluation of soil temperature at different depth horizons to better describe the response of crops to different sowing periods. It is hypothesized that (i) soil temperature varies significantly with sowing date and soil depth, (ii) deeper soil layers provide more stable thermal conditions that influence root activity and crop growth, and (iii) differences in soil temperature across depths contribute to variations in yield, biomass, and resource use efficiency of cotton. Therefore, the objective of this study was to evaluate soil temperature dynamics at different depths (0–30, 30–70, and 70–110 cm) under different sowing dates and to determine their effects on cotton growth, yield, biomass accumulation, and resource use efficiency. This study provides new insights into the role of deep soil thermal conditions in cotton production and offers useful information for developing climate-resilient sowing strategies under changing environmental conditions.

## Materials and methods

### Site description and meteorological conditions

The Institute of Cotton Research, Chinese Academy of Agricultural Sciences (ICR-CASS) experimental station in Anyang, Henan Province, China (36^o^06’ N, 114^o^21’ E) served as the study site for three years (2022–2024). The site has a moderate, temperate continental monsoon climate and is situated at an elevation of 70 m in the North China Plain. The experiment was conducted in a field with continuous cotton monoculture on moderately fertile clay loam soil. The soil was classified according to the FAO and USDA soil classification systems based on its physical and chemical properties. Before the experiment, soil samples were collected from the 0–30 cm layer to determine basic physical and chemical properties. The soil had a bulk density of 1.32 g cm⁻³, pH of 7.8, and consisted of 42% sand, 36% silt, and 22% clay, corresponding to a loam soil according to the USDA textural classification. These values fall within the normal range reported for cultivated agricultural soils. Furthermore, the top 20 cm soil layer had 11.0 g kg^-1^ of organic matter, 0.7 g kg^-1^ of total nitrogen, 10.3 mg kg^-1^ of available phosphorus, and 110.0 mg kg^-1^ of available potassium. An on-site weather station automatically captured meteorological data during the growing season. The soil hydraulic properties were also characterized to support soil moisture analysis. The field capacity (FC) and permanent wilting point (PWP) of the experimental soil were determined using standard pressure plate apparatus at − 33 kPa and − 1500 kPa, respectively. These parameters were used to interpret soil water availability and plant water uptake dynamics. Soil water retention characteristics depend on soil texture and density, which influence water availability for crop growth [39].

**Fig. 1:**
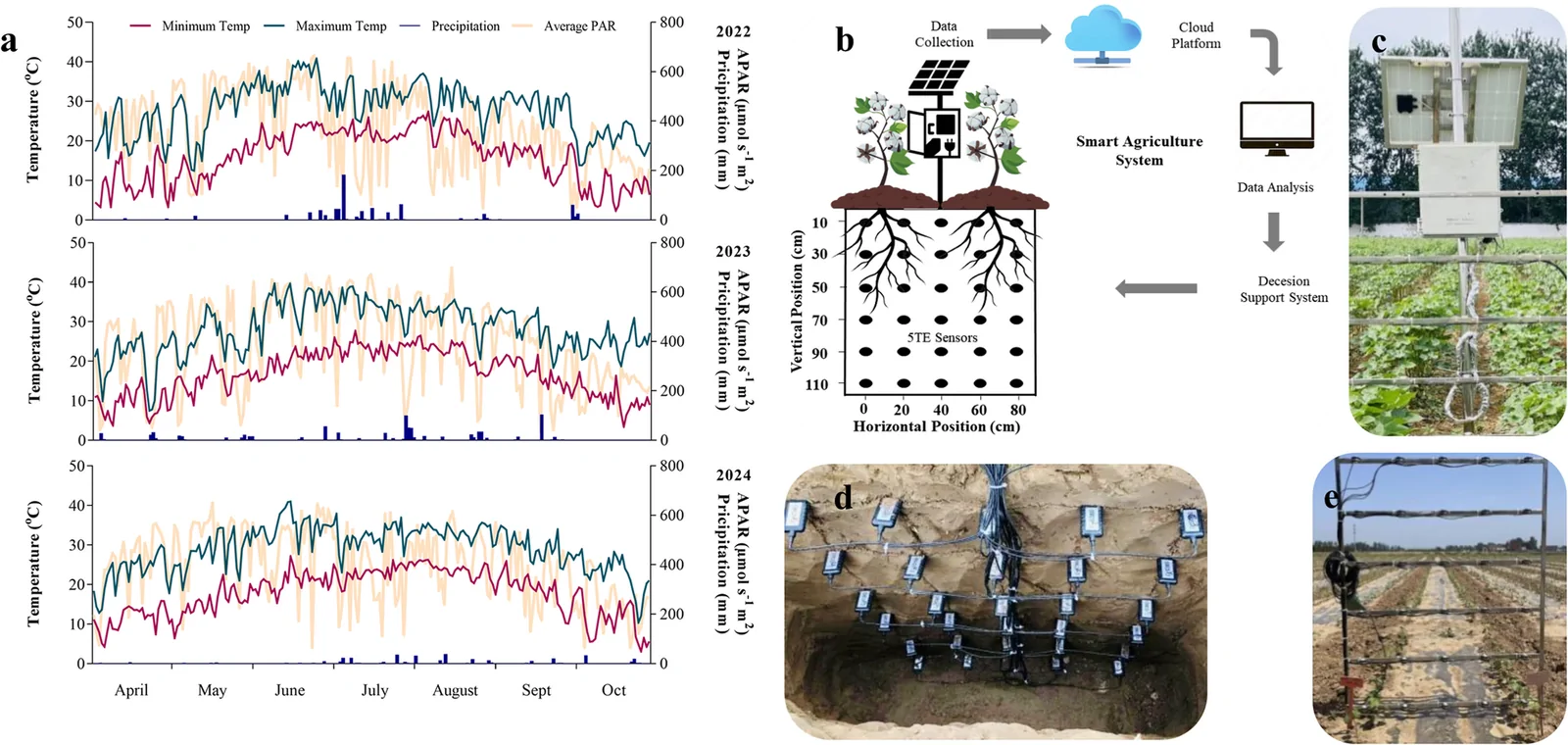
Weather conditions in 2022 to 2024 (**a**), and Smart agricultural sensor networks used in the field experiment: diagram of the smart agricultural sensor network monitoring field light, temperature, and water environment (**b**); protection module, power supply module and control node (**c**); 5TE soil moisture sensors set by the spatial grid method (**d**); linear optical quantum sensor framework (**e**)

Long-term (1991–2020) climatic data obtained from the local meteorological station were used to compare the experimental years with the regional average conditions. Weather conditions varied considerably during the three years (Fig. 1a). Annual cumulative temperature (°C) was calculated as the sum of daily mean air temperatures recorded throughout the growing season. Air temperature was recorded at a standard height of 2 m above ground level at the meteorological station located near the experimental site. In 2024, exceptionally high temperatures were observed, with an annual cumulative temperature of 5172.74 °C. During the cotton growing season (April-October), the mean daily temperature was 23.2 °C, with a maximum of 40.97 °C on June 13, and precipitation totaled 315.9 mm, including a single-day maximum of 37.0 mm on August 9. In 2023, total rainfall was substantially higher at 793.5 mm, with a daily peak of 101.9 mm on September 19. The mean daily temperature was 23.66 °C, with July averaging 29.71 °C and an extreme of 43.12 °C on July 7. In 2022, precipitation totaled 656.6 mm, including 189.7 mm on July 5, the wettest single day across the study period. The highest daily temperature that year was 33.1 °C on June 25, and the mean daily temperature was 21.9 °C.

### Experimental design and plot arrangement

The test variety was the early-maturing cotton cultivar *Chinese Cotton Research Institute 134 (CCRI 134).* Six sowing dates were evaluated (to create clear differences in thermal conditions during early growth while keeping the treatments within the recommended sowing window for the region): April 12 (S1), April 18 (S2), April 24 (S3), April 30 (S4), May 6 (S5), and May 12 (S6). Treatments were arranged in a randomized block design with three replicates. Each plot measured 64 m^2^ (8.0 × 8.0 m). Before sowing, the field was flood-irrigated at 290 m^3^ ha^-1^, and supplemental irrigation of 60 mm was applied in late June. The experiment was conducted under partially irrigated conditions, with irrigation applied when rainfall was insufficient. A buffer space of 1.0 m was maintained between adjacent plots to minimize border and edge effects, with row spacing 60 cm and plant spacing 25 cm. To avoid border effects, plants from the outer rows were excluded and measurements were taken only from the central rows of each plot. A basal fertilizer of 225 kg ha^-1^ P_2_O_5_, and 225 kg ha^-1^ K_2_O was incorporated before planting. Nitrogen fertilizer was applied at a rate of 120 kg N ha⁻¹ (with 50% applied at sowing and the remaining 50% at the flowering stage), which corresponds to the recommended dose for cotton in the region. Standard tillage and weed control were applied uniformly across treatments, and cotton residues were returned to the soil using a ginning machine within one month after harvest. Moreover, pest and disease control was carried out according to local recommended practices, and pesticides were applied when necessary to keep the crop free from serious infestation. Two plants per plot were sampled from the central rows, which is consistent with previous field studies where destructive sampling is limited to avoid disturbing plot uniformity, and the mean values were used for statistical analysis. Destructive sampling was conducted on designated plants only, and the remaining plants were left intact; therefore, final yield measurements were not affected.

### Data collection

#### Investigation of the cotton fertility stage

The cotton fertility period was defined as the interval from seedling emergence to boll opening in 50% of plants. It was divided into five phenological stages: (i) emergence -seedling stage (sowing to 50% seedling leaf expansion), (ii) seedling-squaring stage (S-SQ: emergence to 50% budding), (iii) squaring-flowering stage (SQ-F: bud initiation to 50% flowering), (iv) flowering-boll opening stage (F-B: flowering to 50% boll opening), and (v) boll opening-harvesting (B-H: initial boll opening to final harvest). Table 1 presents the survey results from the cotton growing season under various sowing date treatments in 2022, 2023, and 2024.

**Table 1:** Cotton growth stage and development in 2022, 2023, and 2024

Treatment	Sowing date	Growing stage (m-d/DAS)					
		Emergence-Seedling	Seedling-Squaring	Squaring-Flowering	Flowering-Boll Opening	Boll Opening-Harvesting	Harvest date
Year 2022							
S1	4–12/0	4–22/10	5–17/35	5–31/49	6–27/76	8–20/130	9–29/160
S2	4–18/0	4–28/10	5–17/29	6 − 3/46	6–27/70	8–24/128	9–29/164
S3	4–24/0	5–5/11	5–17/23	6–7/44	7 − 3/70	8–31/129	9–29/158
S4	4–30/0	5–9/9	5–17/17	6–8/39	7 − 4/65	9 − 2/125	9–29/153
S5	5–06/0	5–12/6	5–27/21	6–12/37	7–13/69	9–13/131	9–29/147
S6	5–12/0	5–19/7	5–27/15	6–13/32	7–13/62	9–16/127	9–29/140
Year 2023							
S1	4–12/0	4–19/7	5–19/37	6–12/61	7–7/86	8–30/140	10 − 9/150
S2	4–18/0	4–28/10	5–19/31	6–11/54	7–8/79	8–31/128	10 − 9/174
S3	4–24/0	5 − 2/8	5–19/25	6–11/48	7 − 6/73	8–24/122	10 − 9/168
S4	4–30/0	5–7/7	5–19/19	6–14/45	7–10/71	8–31/123	10 − 9/163
S5	5–06/0	5–12/6	5–19/13	6–18/43	7–13/68	9 − 2/119	10 − 9/126
S6	5–12/0	5–18/6	6 − 2/21	6–23/42	7–18/67	9 − 4/115	10 − 9/120
Year 2024							
S1	4–12/0	4–20/8	5–13/31	5–26/44	6–24/73	8–14/124	10 − 9/180
S2	4–18/0	4–26/8	5–13/25	5–26/38	6–23/66	8–14/118	10 − 9/119
S3	4–24/0	4–30/6	5–13/19	6 − 1/38	6–29/66	8–21/119	10 − 9/168
S4	4–30/0	5–7/7	5–31/31	6 − 4/35	7 − 1/62	8–25/117	10 − 9/162
S5	5–06/0	5–13/7	5–31/25	6–11/36	7–9/64	8–30/116	10 − 9/156
S6	5–12/0	5–20/8	5–31/19	6–14/33	7–13/62	9 − 2/113	10 − 9/120

#### Biomass measurement

Biomass sampling was conducted at approximately 15-day intervals during each growing season: at 41, 55, 71, 84, 100, 113, 127, and 144 days after sowing (DAS) in 2022; at 40, 55, 72, 87, 102, 115, 126 and 143 DAS in 2023; and at 40, 58, 71, 86, 97, 108, 122, and 140 DAS in 2024. At each sampling, two plants per plot were randomly uprooted and partitioned into roots (below the cotyledonary node), stems and leaves (aboveground vegetative organs), and reproductive organs. Roots were carefully washed with running water over a fine mesh to avoid loss of fine roots during cleaning. Samples were oven-dried at 105 °C for 30 min to halt metabolism and subsequently at 80 °C for 48 h to constant weight.

#### Cotton yield and its components

In early October, the number of bolls was counted in the two central rows of each plot. Manual harvesting was conducted three times at 10-day intervals. Seed cotton yield was adjusted to a standard moisture content of 8% before conversion to yield per hectare. Before each harvest, 100 representative bolls were collected. Seed cotton yield (SCY) and lint yield (LY) were measured after harvest, and Single boll weight (SBW) an measured in g plant⁻¹ and lint percentage (LP) were subsequently calculated. Seed cotton samples were ginned using a laboratory gin to separate lint and seed before determining LP.

#### Canopy photosynthetically active radiation acquisition and measurement

Photosynthetically active radiation (PAR) was measured using a linear quantum sensor (LI-191SA, LI-COR, Lincoln, NE, USA) connected to a data logger (LI-1400, LI-COR). Thirty sensors were mounted on a metal frame in a uniform spatial grid with 20 cm spacing (Fig. 1e). The grid consisted of six rows (with a 100 cm vertical spacing, starting 10 cm above the soil surface) and five columns (with an 80 cm horizontal spacing across the cotton rows). A total of 30 sensors were used per treatment, distributed across all replications, resulting in treatments × replications × depth sensors in the entire experiment. PAR was recorded simultaneously on each plant sampling date in the two central rows of each plot, with sensors positioned parallel to the cotton rows and fixed in place. Incident PAR was continuously measured and logged after canopy establishment. Furthermore, Leaf area index (LAI) was measured periodically to evaluate canopy development and its effect on radiation interception.

#### Measurement of microclimate monitoring

A cloud-based platform was established for data collection, transmission, and storage via the Internet (Fig. 1b-c). During the cotton growth and development season, 5 TE sensors [40] were used to monitor soil volumetric water content and temperature (Fig. 1d). The 5TE sensors (Decagon Devices, USA) measure volumetric water content based on dielectric permittivity, with an accuracy of ± 3%, and were factory-calibrated and further validated under field conditions prior to installation. Before installation, sensors were calibrated using the gravimetric soil-drying method. To minimize disturbance, plots were selected before planting, and sensors were positioned at the plot center following the spatial grid method [41–43] Sensors were vertically inserted into 80 × 100 cm soil profiles at 20 cm intervals both horizontally and vertically (Fig. 1d), resulting in 30 insertion points per treatment. Dielectric permittivity (ℇa) and soil temperature were recorded hourly, and soil water content was estimated using the following equation [44, 45].

1 $$SWC=\:4.3\times{10}^{-6}\varepsilon^{2}a-5.5\times{10}^{-4}\varepsilon^{2}a+2.92\times{10}^{-2}\varepsilon a-5.3\times{10}^{-2}$$

### Data processing

#### Methods for measuring water consumption

Total cumulative water consumption was calculated by integrating hourly soil moisture changes across the spatial grid. Grid values represent the spatially distributed volumetric water content at each sensor node, which were aggregated using Simpson’s 3/8 numerical integration to estimate total soil water volume and its temporal depletion. A spatial grid (i, j) file was generated from data collected by 30 sensors using the spatial grid method for each sowing date treatment, where *i* and *j* denote the abscissa and ordinate, respectively. Simpson’s 3/8 rule was expanded and used to determine the volumetric water content for each grid. The equations used in this procedure are presented below [45]:

2 $$\:{A}_{i}=\:\frac{3\varDelta\:X}{8}\:\left[{G}_{i,1}\:+3{G}_{i,2}\:+2{G}_{i,4\:\:}+\dots\:+2{G}_{i,ncol-1}+\:{G}_{i,ncol}\right]$$

3 $$\:\:V\:\approx\:\:\frac{3\varDelta\:Y}{8}\:\:\left[{A}_{i\:\:\:}+\:\:3{A}_{2\:\:\:}+3{A}_{3}\:+2{A}_{4\:\:\:}+\dots\:+2{A}_{ncol-1}+\:{A}_{ncol}\:\:\right]$$

4 $$\:\:{Grid}_{ij}=\:\frac{V}{area}$$

The coefficient vector was applied in the calculation, where *G*_*i, j*_ denotes the depth at grid point (*i*,* j*) and *ΔX* and *ΔY* represent the column and row spacing of the grid file, respectively. *A*_*i*_ denotes the *i*th cross-sectional area and *V* the corresponding total water volume. *Grid*_*i, j*_ denotes the integral average of soil water content within each grid element, and *area* refers to the soil sampling profile area.

The cumulative water consumption (WC), calculated as seasonal water use (mm season⁻¹), of a crop population was calculated as follows.

5 $$WC=\sum\limits_{i=1}^{n}{(Grid}_{n}-\left({Grid}_{n+1}\right)$$

Where *n* is the order of the grid files and the number of hours when sampling. Data in the range of *WC* less than 0.0 mm h^− 1^ due to increased soil moisture and *WC* greater than 0.1 mm h^− 1^ due to drastic changes in moisture due to extreme weather were excluded [42, 46].

#### Effective accumulated soil temperature

The four-point method, which involves averaging temperature data obtained at 2:00, 8:00, 14:00, and 20:00, was used to determine the daily average soil temperature [45]. The daily average temperature for crop and root growth was subtracted from the biological threshold temperature of 10 °C, which is thought to be the lowest needed to support regular physiological activities [47]. The cumulative sum of these daily effective temperature readings over time was then calculated to determine the effective accumulated soil temperature (EAT) [48, 49]. EAT was calculated separately for each soil depth.

#### Growing degree days calculation

Growing degree days (GDD) was used to quantify heat accumulation of air during the cotton growing season, defined as the sum of daily mean temperatures exceeding the lower biological threshold of 15 °C for cotton growth [49] and the Heat Unit Index (HTU) was used to quantify crop efficiency in utilizing GDD during the growing season [50, 51]. This threshold mainly represents shoot growth, although root activity may occur at slightly lower temperatures. When the daily mean temperature was below the base temperature, the GDD value was set to zero. GDD and HTU were calculated using the following formulas:

6 $$GDD=\sum\limits_{t=1}^{N}{GDD}_{t}\:=\left\{\begin{array}{c}\:\:\:\:\:\:\:0\:\:\:\:\:\:\:\:\:\:\:\:\:\:({T}_{av}\le\:\:{T}_{base}\:)\\\:{T}_{av\:}-\:{T}_{base\:\:\:}({T}_{av\:}\:\le\:{T}_{base}\:\end{array}\right.$$

7 $$HTU=GDD\times Actual\:daily\:sunshine\:hour$$

Where $\:{T}_{av}$ is the average daily temperature (°C) and $\:{\mathrm{T}}_{\mathrm{b}\mathrm{a}\mathrm{s}\mathrm{e}}$ = 15 °C. The daily mean temperature was calculated using the four-point method, i.e., the average of measurements taken at 2:00, 8:00, 14:00, and 20:00 [46].

#### Change in cotton biomass measurement

Biomass refers to total aboveground dry matter unless otherwise stated. A nonlinear logistic growth model was applied to evaluate the effect of effective accumulative soil temperature (EAT) on cotton biomass, expressed as [41]:

8 $$\:Y\:=\:\frac{A}{1+b{e}^{-kt}}$$

where *Y* is biomass, *A is* the theoretical maximum biomass value, *t* indicates the effective accumulated soil temperature, and *b* and *k* are model parameters.

#### Resource utilization efficiency

Accumulated air temperature use efficiency (TPE, kg.ha^-1^°C^-1^), heat use efficiency (HUE, kg.ha^-1^°C^-1^) [16, 52], radiation use efficiency (RUE, kg.ha^-1^MJ^-1^m^2^) [53, 54], soil water use efficiency (WUE, kg.ha^-1^mm^-1^), and accumulated soil heat productive efficiency (PE_soil_, kg. ha^-1^◦C^-1^). PEsoil index was developed in this study to evaluate the relationship between soil temperature and crop performance [55, 56]; which are calculated using the following formulas:

9 $$TPE=\frac{\varDelta\:Biomass}{GDD}$$

10 $$HUE=\frac{Seed\:Cotton\:Yield}{GDD}$$

11 $$RUE=\frac{Biomass}{PAR}$$

12 $$WUE=\frac{Lint\,Yield}{WC}$$

13 $${PE}_{soil}=frac{Lint\,Yield}{EAT}$$

#### Software

Data processing was performed in Microsoft Excel 2019 (Microsoft, Bothell, WA, USA) and Stata 16 (Stata Corp LP, College Station, Texas, USA). Graphs were prepared using PowerPoint 2019 and GraphPad Prism 8, which was also used for fitting the logistic model. The spatial distribution of soil water consumption by soil profile and its relationship with seed cotton yield were prepared using Golden Software Surfer 28. Spearman’s correlation and analysis of variance (ANOVA) were carried out in Origin 24 (Origin Lab, Northampton, MA, USA) and SPSS 25 (SPSS Inc., Chicago, IL, USA), respectively. PCA was performed using R software (version 4.3.3). Treatment means were compared using the least significant difference (LSD) test at the 0.05 probability level.

## Results

### Seasonal variation and stability of weather conditions from 2022 to 2024

The meteorological patterns of the years 2022, 2023 and 2024 are unique, especially in terms of temperature, precipitation and humidity, displaying environmental conditions peculiar to each of the three years (Fig. 1a). The rainfall regime in 2022 features a high frequency of extreme rainfall events combined with few intermittent precipitation events that occur throughout the year, indicating the predominance of intensity rainfall. Temperature was strongly coupled with the warmer and shallower water during major warm episodes, with large seasonal variations. The humidity was very variable, with notable drops corresponding to droughts (Fig. 1a).

Year 2023 has prevailed with climatic conditions close to 2022 but with abated extremes. The climate was also less variable, with a lower temperature range and seasonal amplitude. The amplitudes of climatic variability were smaller with less variation in temperature and less seasonal stratification. Despite continuing the same reversing precipitation variability, it was more normalized and consistent compared to 2022 (Fig. 1a), leading to a lower occurrence of heavy rainfall events. The humidity level in 2023 was also found to be less volatile than in 2022 with fewer severe reductions and appears to provide a more predictable and uniform amount of moisture at any point in the year (Fig. 1a). All these observations imply generally more stable and conducive climatic conditions over 2023 compared to 2022, and hence crop growth is expected to be more supportive in 2023 compared to 2022.

By 2024, the weather patterns had become even more stable (Fig. 1a). Differences in temperature were not dramatic, and the year was the one with minimal seasonal change, meaning it is a milder climate. The precipitation was spread more smoothly, and there were fewer tons of intense spikes in the rainfall than in 2022 and 2023, which indicated drier conditions altogether. Another minimum fluctuation was observed in humidity, where the yearly moisture level was constant. This indicates that 2024 had the most consistent and predictable weather conditions among the three years, with fewer extreme weather events, which may have positively impacted the resource allocation for crops, offering a stable environment for growth.

Seasonal rainfall differed markedly among years, totaling 656.6 mm in 2022, 793.5 mm in 2023, and 315.9 mm in 2024, indicating strong inter-annual variability in precipitation distribution. The highest rainfall occurred in 2023, whereas 2024 was comparatively dry (Fig. 1a). Relative humidity also varied among years, with higher fluctuations observed in 2022 and more stable conditions in 2023–2024, as shown in Fig. 1a. Analysis of variance showed significant differences among years for temperature and precipitation variables (*P* < 0.05), confirming that the three growing seasons represented different climatic conditions.

### Relationship between biomass accumulation and effective accumulated temperature across sowing dates

**Fig. 2:**
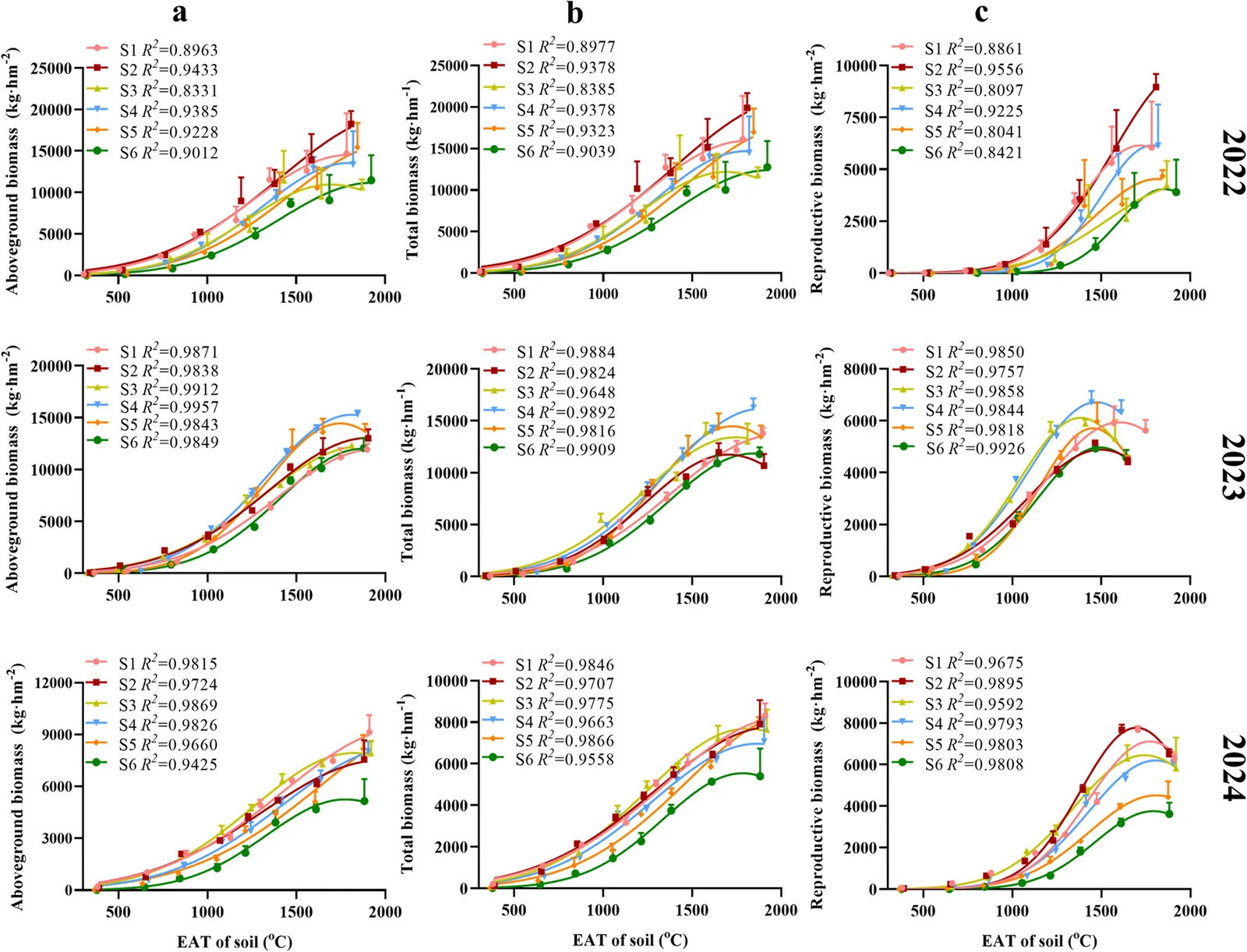
Relationship between the accumulation of aboveground biomass (**a**), total biomass (**b**), and reproductive biomass (**c**), and the Effective Accumulated Temperature (EAT) of soil in different sowing date treatments in 2022, 2023, and 2024. A logistic growth model was used to fit the curves

The ability of cotton to accumulate biomass was strongly influenced by sowing date, which governed the extent to which plants could harness available thermal resources during the growing period (Fig. 2a-c). Across all three years (2022–2024), the relationship between effective accumulated temperature (EAT) and biomass accumulation followed a logistic growth curve pattern, and the logistic growth model provided an excellent fit in most cases (Fig. 2). However, model conformity, as reflected in the coefficients of determination (R²), varied considerably among sowing dates. Earlier sowings (S1 and S2) consistently demonstrated superior alignment between EAT and biomass production (aboveground, total biomass, and reproductive biomass), indicating efficient utilization of thermal units, whereas intermediate sowings (S3, S4) showed moderate conformity and later sowings (S5, S6) often lagged, particularly under the variable climatic conditions of 2022. In subsequent years, model stability improved markedly, with R² values exceeding 0.95 across all sowing dates (Fig. 2a-c).

For aboveground biomass, the efficiency of thermal resource capture was highest under early sowing (Fig. 2a). In 2022, S2 exhibited the strongest relationship (R² = 0.9433), followed by S1 (R² = 0.9376), while the weakest association was recorded in S3 (R² = 0.8331). Later sowings (S4–S6) showed moderate fits, reflecting partial inefficiency in translating accumulated temperature into vegetative biomass. In 2023, a year characterized by more favorable and consistent climatic conditions, all sowing dates achieved very high conformity (R² > 0.98), with S2 (R² = 0.9957) and S3 (R² = 0.9956) demonstrating near-perfect alignment with the logistic curve (Fig. 2a). This stability persisted in 2024, where early sowings again led (S2, R² = 0.9869) and later sowings such as S5 displayed relatively weaker, though still strong, conformity (R² = 0.9425) (Fig. 2a).

For total biomass, a similar sowing-date effect pattern was observed, as biomass differed significantly among sowing dates (*p* < 0.05) (Fig. 2b). In 2022, early sowings (S2, R² = 0.9378; S1, R² = 0.9263) maximized resource-use efficiency, whereas S3 recorded the lowest conformity (R² = 0.8385). Moreover, in 2023, thermal utilization across all sowings was highly efficient (R² = 0.9842–0.9909), with S2 achieving the best fit (R² = 0.9909). In 2024, a similar pattern was observed, with early sowings demonstrating higher conformity (S2, R² = 0.9866), while the weakest association was observed in S6 (R² = 0.9558) (Fig. 2b).

For reproductive biomass, the variability among sowing dates was even more pronounced, particularly in 2022 (Fig. 2c). S2 exhibited the strongest correlation with thermal accumulation (R² = 0.9556), while late sowings such as S5 (R² = 0.8041) and S6 (R² = 0.8859) displayed the weakest conformity. Intermediate sowings, S3 (R² = 0.8928) and S4 (R² = 0.9054), also showed moderate relationships. In contrast, the stability of reproductive biomass accumulation improved substantially in 2023, where all sowing dates demonstrated very high conformity (R² = 0.9844–0.9926), with S1 achieving the best fit (R² = 0.9926). This pattern continued in 2024, where the logistic model explained reproductive biomass dynamics robustly across sowings, ranging from R² = 0.9592 (S4) to 0.9895 (S1) Fig. 2c).

### Relationship between effective soil temperature across sowing dates

**Fig. 3:**
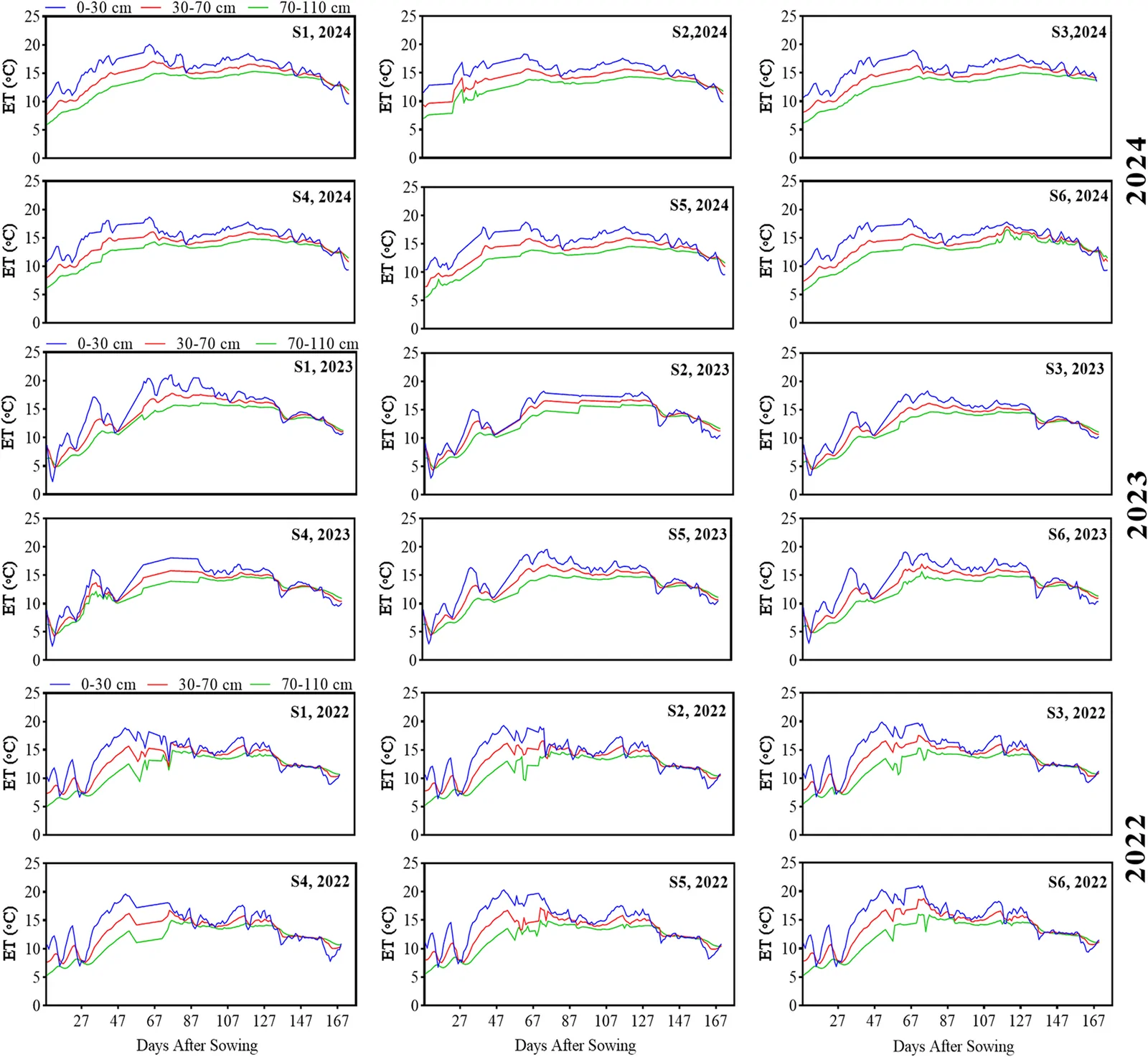
Variation in the effective soil temperature (ET) by sowing date treatment and by depth (upper 0–30 cm), middle (30–70 cm), lower (70–110 cm) with DAS (Days After Sowing) in cotton during cotton growth and development in 2022,2023, and 2024. The ET is the original temperature minus the lower growth limit temperature of cotton of 10 ℃

Effective Soil Temperature (ET) data recorded in the three different soil depths (0–30 cm, 30–70 cm, and 70–110 cm) during the years 2022, 2023, and 2024 showed a considerable difference in the accumulation of the temperature of the soil at different sowing dates (S1 through S6) (Fig. 3a-c). A significant interaction was observed among year, sowing date, and soil depth (*p* < 0.05).

At a depth of 0–30 cm (corresponding to the blue line), there was an evident positive trend of ET values with increasing sowing dates. The ET was relatively lower at the first sowing date (S1 and S2) and was on the rise to S6, with the highest values at later sowing dates. This trend was followed in 2023 and 2024, with the higher ET values being noted at later sowing dates (S5 and S6), and in 2024, the ET was relatively stabilized, but it still showed a steady increase with the older sowing dates. This depth continuously registered the best ET values in all the years, but particularly in S4 and S6, all three years recorded high increases (Fig. 3a-c).

At the depth of 30–70 cm (which is represented by the red line), ET similarly rose with later sowing dates, although it was more variable than at the depth of 0–30 cm. There was a significant rise in ET within the S3 and S4 in 2022, which means that there was a responsive warming of the soil as the season advanced to sowing (Fig. 3a-c). This was observed into 2023 and 2024 with the ET registering greater values at the later sowing dates, specifically between S4 and S6. Nevertheless, the ET in this depth was always lower compared to the depths of 0–30 cm, and more significantly variable in 2023 and 2024 than in 2022.

The slowest and most gradual average increase in ET over a period of time was seen in the 70-110 cm depth (represented by the green line). In 2022, ET was constant, but only slight gains were observed between dates of sowing (Fig. 3a-c). The 2023 and 2024 data values denoted that the gradual increase in ET at this depth continued, and the values deviated slightly after S4 in the respective years. The ET in this depth was, however, always low as compared to the depths of 0-30 cm and 30-70 cm (Fig. 3a-c). The pattern in all years indicated that lower layers of the soil stored heat at a lower rate, and the least difference between sowing dates, particularly at later sowing dates (S5 and S6).

### Trends in effective accumulative soil temperature at different soil depths (2022–2024)

**Fig. 4:**
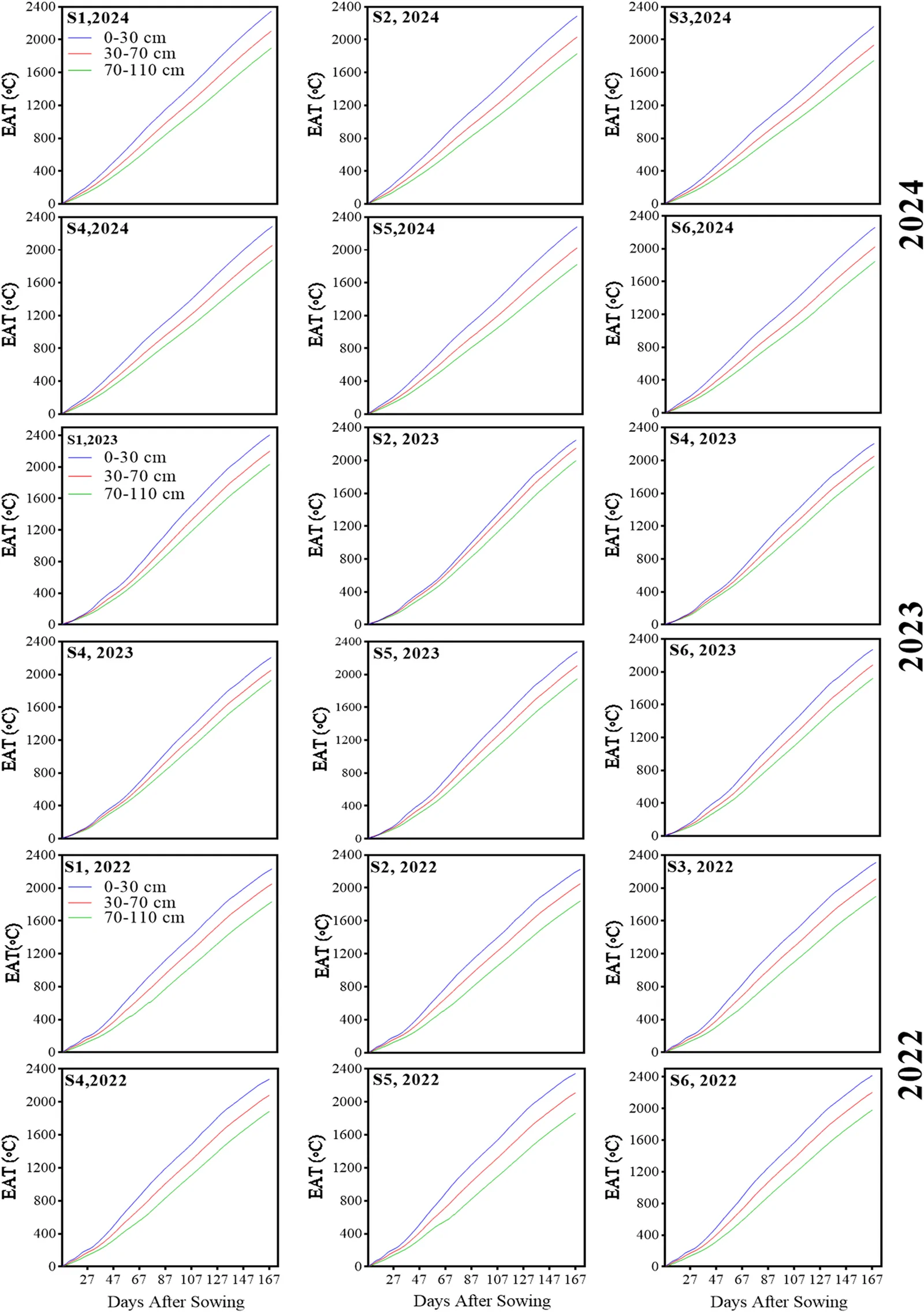
Change in the effective accumulative soil temperature (EAT) by soil profiles (upper 0–30 cm), middle (30–70 cm), lower (70–110 cm) with DAS (Days After Sowing) in different sowing date treatment during cotton growth and development in 2022, 2023, and 2024

The projections of Effective Accumulative Soil Temperature (EAT) of the years 2022, 2023, and 2024 at varying soil depths (0–30 cm, 30–70 cm, and 70–110 cm) exhibit different trends across the various stages (S1 to S6) (Fig. 4a-c). EAT at deeper soil layers showed a significant positive relationship with yield (*p* < 0.05). In the case of the depth of 0–30 cm (which was represented by the blue line), the increase in EAT has been steady and consistent throughout the three years. The EAT at that depth also experienced a significant increase in 2022, and the trend persisted in 2023, which was an efficient buildup of temperature. By 2024, the increase in EAT at this depth began to level off, which indicates that by that point, the resource allocation approach at the shallowest layer of the soil was at its optimal level.

The EAT had more variations in the three years at a depth of 30–70 cm (drawn by the red line). The red line recorded an early rise in EAT in 2022, although there was some variation in 2023, implying a less stable allocation of resources at this depth. Nevertheless, a more pronounced and steady increase was also observed in the EAT by the year 2024, which suggests that there were changes in resource allocation to maximize the accumulation of heat at this mid-depth point (Fig. 4a-c). This layer exhibited the highest range of variation in the data, and it showed that the resource allocation could have been more often revised to reflect varying circumstances.

Furthermore, the EAT had the least increase in the 70–110 cm depth (that is depicted by the green line) when compared to the other depths, with a gradual increase over the years. In 2022, the green line showed a slower growth of temperature, and the same trend was observed in 2023 and 2024 (Fig. 4a-c). The green line was more stable and moderate than the other depths, and this indicates that the resource allocation at this deeper level was more conservative, implying slower allocation of temperature.

### Optimizing seed cotton yield through sowing date and resource allocation strategies

**Fig. 5:**
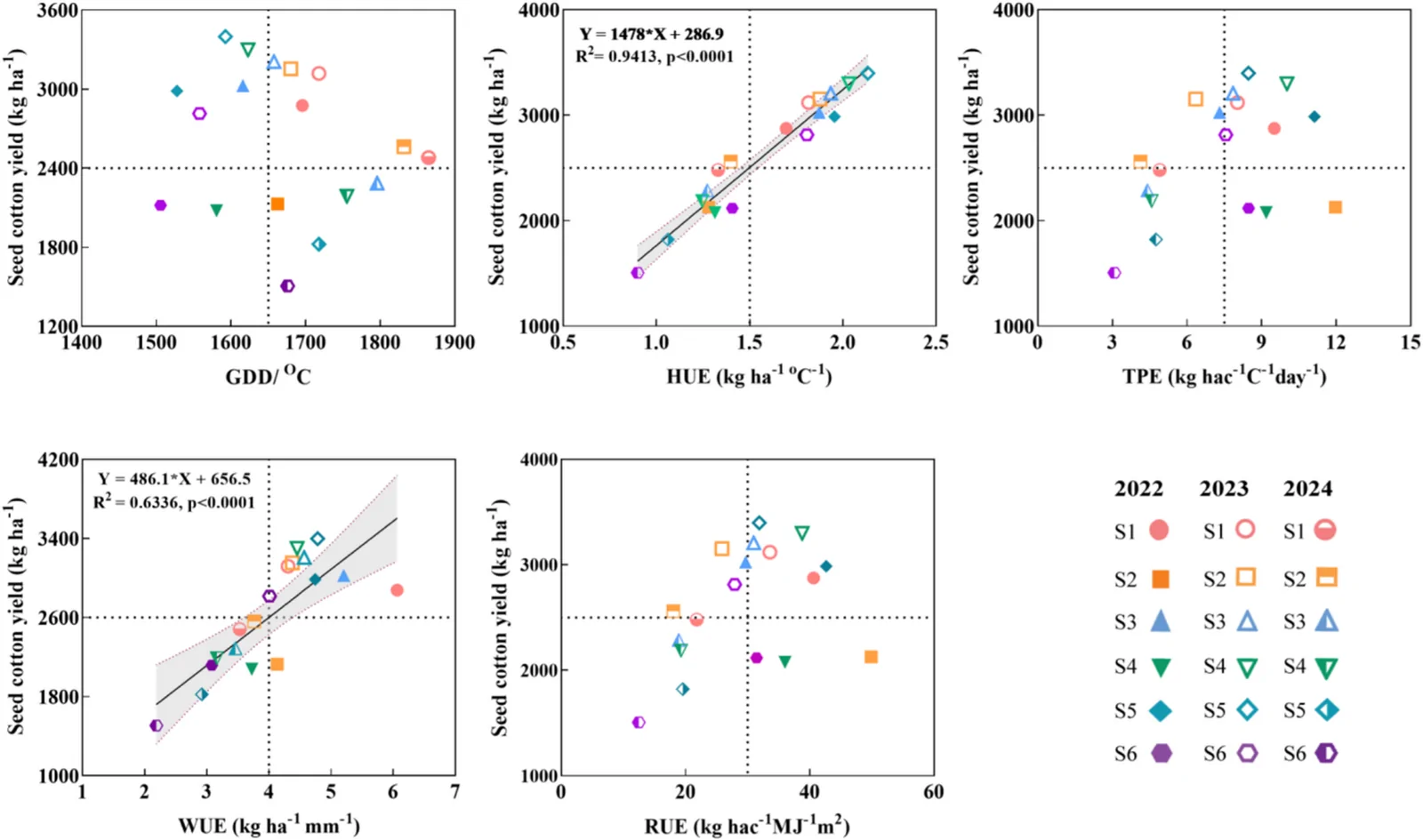
Linear relationship between seed cotton yield and Growing Degree Days (GDD), Heat Use Efficiency (HUE), Accumulated Air Temperature Efficiency (TPE), Water Use Efficiency (WUE), Radiation Use Efficiency (RUE). The shaded area represents the 95% confidence interval for the best-fit line.

The seed cotton yield was measured at sowing dates (S1 to S6) during 2022, 2023, and 2024, with resource allocation (in terms of Growing Degree Days (GDD), Heat Use Efficiency (HUE), Accumulated air Temperature use Efficiency (TPE), Water Use Efficiency (WUE), and Radiation Use Efficiency (RUE)) having an impact on yield per year (Fig. 5a-e). Table 2 shows the difference between cotton yield and yield components for each sowing date treatment among three consecutive years. Yield was significantly affected by the interaction between year and sowing date (*P* < 0.05). Although early sowing showed higher efficiency indices, the highest yield was not always obtained in the same treatment. The findings showed that the seed cotton yield was significantly varied in response to the varying sowing dates, which was associated with the changing resource allocation strategies within the three years.

**Table 2:** Cotton yield and yield components for each sowing date treatment in 2022, 2023, and 2024

Sowing date	Seed cotton yield (Kg. ha^− 1^)	Lint percentage (%)	Lint Yield (Kg. ha^− 1^)	Single boll weight (g)
Year 2022				
S1	2876.3 a	36.547 a	1051.5 a	5.6077 a
S2	2129.4 b	35.397 c	754.2 b	5.7404 a
S3	3025.9 a	36.742 a	1112.3 a	5.6980 a
S4	2077.2 b	35.674 bc	742.4 b	5.7978 a
S5	2986.3 a	36.222 ab	1081.4 a	5.7813 a
S6	2118.4 b	35.310 c	749.2 b	5.7886 a
Year 2023				
S1	3119.9 c	37.632 a	1174.1 c	11.939 abc
S2	3153.4 c	37.502 ab	1182.4 c	11.816 bc
S3	3208.3 bc	37.688 a	1209.1 bc	11.675 c
S4	3298.6 ab	37.729 a	1244.8 ab	12.211 ab
S5	3398.5 a	37.482 ab	1273.7 a	12.189 ab
S6	2815.5 d	36.872 b	1038.0 d	12.278 a
Year 2024				
S1	2481.2 a	34.587 b	858.04 ab	9.4682 a
S2	2561.7 ab	35.840 a	917.75 a	9.4848 a
S3	2286.5 bc	34.425 b	786.97 bc	9.5369 a
S4	2188.2 c	34.059 bc	744.88 c	9.7068 a
S5	1823.1 d	33.959 bc	619.80 d	9.7314 a
S6	1506.8 e	32.996 c	496.31 e	9.8907 a
Analysis of Variance				
Year	***	***	***	***
Sowing date	***	***	***	0.057
Year*Sowing date	***	***	***	0.914

According to the LSD test, different letters indicate significant differences at *P* ≤ 0.05. *, **, *** represent significance at *P* ≤ 0.05, *P* ≤ 0.01, and *P* ≤ 0.001, respectively. Means within each column and year, followed by different lowercase letters (a-e), indicate statistically significant differences among treatments at *P* ≤ 0.05

The correlation of seed cotton yield and GDD was not similar across the years. There was a decrease in yield because of earlier sowing dates (S1 and S2) as GDD increased, where a significant yield improvement was observed at later sowing dates (S5 and S6). In particular, S5 and S6 were the highest-yielding seed cotton because of greater thermal accumulation at these later stages. In 2022, the highest yield occurred in S3, whereas in other years higher yields were observed in later sowing dates. In 2023, there were slight improvements in the GDD of sowing dates S1 and S2, where higher yields were found than in 2022, although the pattern of increasing yields with later sowing dates (S5 and S6) persisted. The findings in 2024 showed a significant increase in all the sowing dates, especially S5 and S6, where the GDD values are increased as compared to the previous years (Fig. 5a). This means that the resource distribution approach to the thermal accumulation process also became better, and the output of seed cotton increased, especially at later dates of sowing. The findings of all years clearly indicate that the later the sowing dates (S5 and S6) were, the better the conditions under which GDD was amenable, and this gave the greatest yields in the particular year.

There was evident year-to-year improvement in the relationship between seed cotton yield and HUE, especially in the later sowing dates. Sowing dates S1 and S2 yielded significantly lower with lower HUE values, and S5 and S6 yielded the highest seed cotton, which was a positive indication of an improved harvest efficiency at the end of the season in 2023 (Fig. 5b). The correlation between HUE and seed cotton yield in 2023 was higher, and it indicates a more refined strategy of resource allocation. The refined resource allocation was the best in 2024, with S5 sowing dates showing the highest HUE and seed cotton yield. The year-to-year change shows that there has been a steady increase in resource usage efficiency, especially in late sowing dates (S5 and S6), which were advantaged by a better thermal and radiation environment, and therefore, had a higher HUE and, consequently, higher yield.

There was a statistically significant difference in the WUE versus seed cotton yield over the years. Early sowing dates (S1 and S2) were found to have worse WUE and lower yields in 2022, indicating that the resource allocation of water was not optimized to grow in the early season. S3 and S4 dates were intermediate in terms of improvement in the sowing dates, and the later sowing dates (S5 and S6) had the highest WUE and yield, indicating the most efficient use of the water resources (Fig. 5d). 2023 was no exception to the pattern, except that the greater the sowing date, the more the ratio between WUE and yield was lower. In 2024, WUE was significantly refined for all the sowing dates. Although S1 and S2 recorded some improvement in WUE when compared to past years, the later stages of sowing (S5 and S6) recorded the highest WUE, hence the high seed cotton yields.

Correlation of TPE and yield of seed cotton indicates that there is an improvement in yield, as higher TPE values. A significantly lower average date of early sowing (S1 and S2) than late (S5 and S6) was observed in 2022 (Fig. 5c). Particularly, the delayed sowing dates (S5 and S6) had higher TPE measurements, which led to higher yields of seed cotton. The trend kept up in 2023 and 2024, with S5 and S6 remaining with the best TPE and the highest yield of seed cotton. These findings can be used to propose that the positive development and performance of seed cotton with the application of TPE were vital, particularly in the later sowing dates, as the resources of thermal productivity were better enhanced.

The correlation between RUE and seed cotton yield also showed the increased results over the years. The first and second sowing date (S1 and S2) reduced the values of RUE and the yield as well. Best results were recorded in sowing the date S5 and S6 whereby RUE was maximized because the resources were allocated more towards the use of solar radiation (Fig. 5e). The association between RUE and seed cotton yield was reinforced in 2023, as the sowing dates (S5 and S6) had uniformly higher RUE and yield than the yield in 2022. Values of RUE were greater in all sowing dates, and S5 and S6 still dominated earlier sowing dates in values of RUE and yield. This means that the later the date of sowing, the better, especially in seed cotton production as resource allocation on the utilization of radiation became more efficient.

### Spatial patterns of soil water consumption and cotton yield over three years

**Fig. 6:**
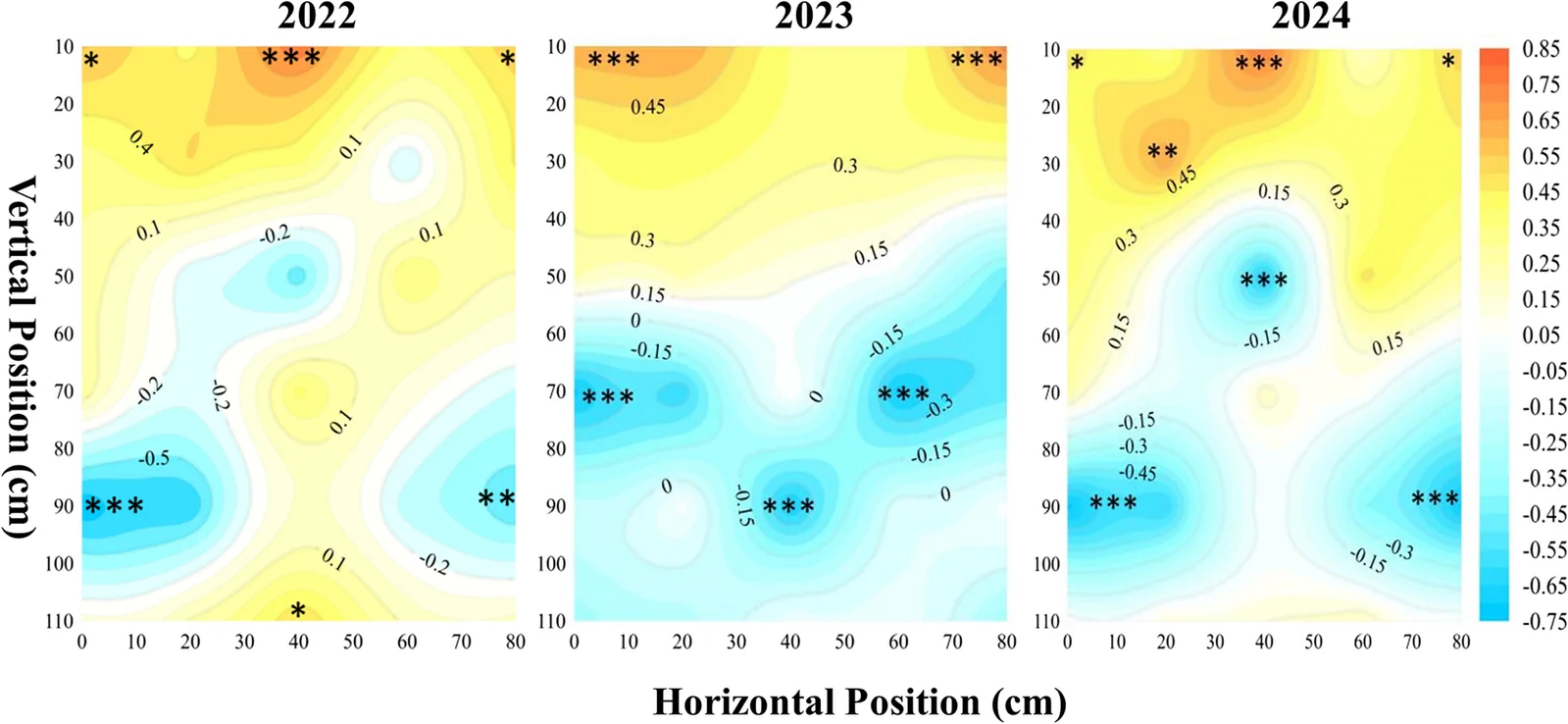
Relationship between soil water consumption distributed throughout the soil profile and seed cotton yield. (* indicates *p* < 0.05, ** indicates *p* < 0.01, and *** indicates *p* < 0.001)

The contour plots indicate the correlation of soil water intake in the entire soil profile and the yield of seed cotton in the years 2022, 2023, and 2024. The data show that the consumption of soil water has a great spatial effect on seed cotton production, and the pattern of positive and negative correlation varied in the three years (Fig. 6).

In the current year, 2022, the correlation between the soil water consumption and seed cotton yield was predominantly negative (x = 40–60, y = 70–90), and thus, when water was over-consumed, it led to low cotton yields. The regions of high negative correlation (three asterisks), especially in the deeper soil layer suggest that superficial water absorption in the regions was negative to the growth of cotton (Fig. 6). Conversely, high layers of soil exhibited weak positive, in which moderate water consumption yielded positive yield results though the total relationship existing between water consumption and seed cotton yield was not optimal and had little areas of positive correlation.

As of 2023, the general trend has changed to more balanced consumption of water. Negative correlations were observed in some of the deeper layers (x = 40–60, y = 60–80), but were not as widespread as in 2022. The statistically significant regions (in which the asterisks are used) of the negative correlation were smaller, which means that the consumption of the soil water was better in the current year than in the past year (Fig. 6). The regions of positive correlations were widened in 2023, especially in the middle profile (x = 30–50, y = 40–70), where water utilization was better adjusted to cotton growth, and resulting in increased yield in the areas. These enhancements indicate that the distribution of resources to the soil water in 2023 was more optimal than in 2022, which meant that the adverse effect of excessive water penetration into the lower layers of the soil profile was mitigated.

The trends of correlation were even more significant in 2024, especially the positive correlation between water consumption and seed cotton yield. The zones of negative correlation (blue areas) became significantly lower, particularly in the layers (x = 40–60, y = 50–70), where water consumption had been particularly troublesome in the previous years (Fig. 6). The statistically significant areas revealed higher positive correlation between water use and cotton yield in the middle and upper soil profile layers (x = 30–50, y = 30–60). By the year 2024, there was greater soil water consumption efficiency in the profile, and therefore the positive association made between water use and seed cotton yield was better in relation to the two previous years.

### PCA and correlation analysis of key variables affecting cotton production

**Fig. 7:**
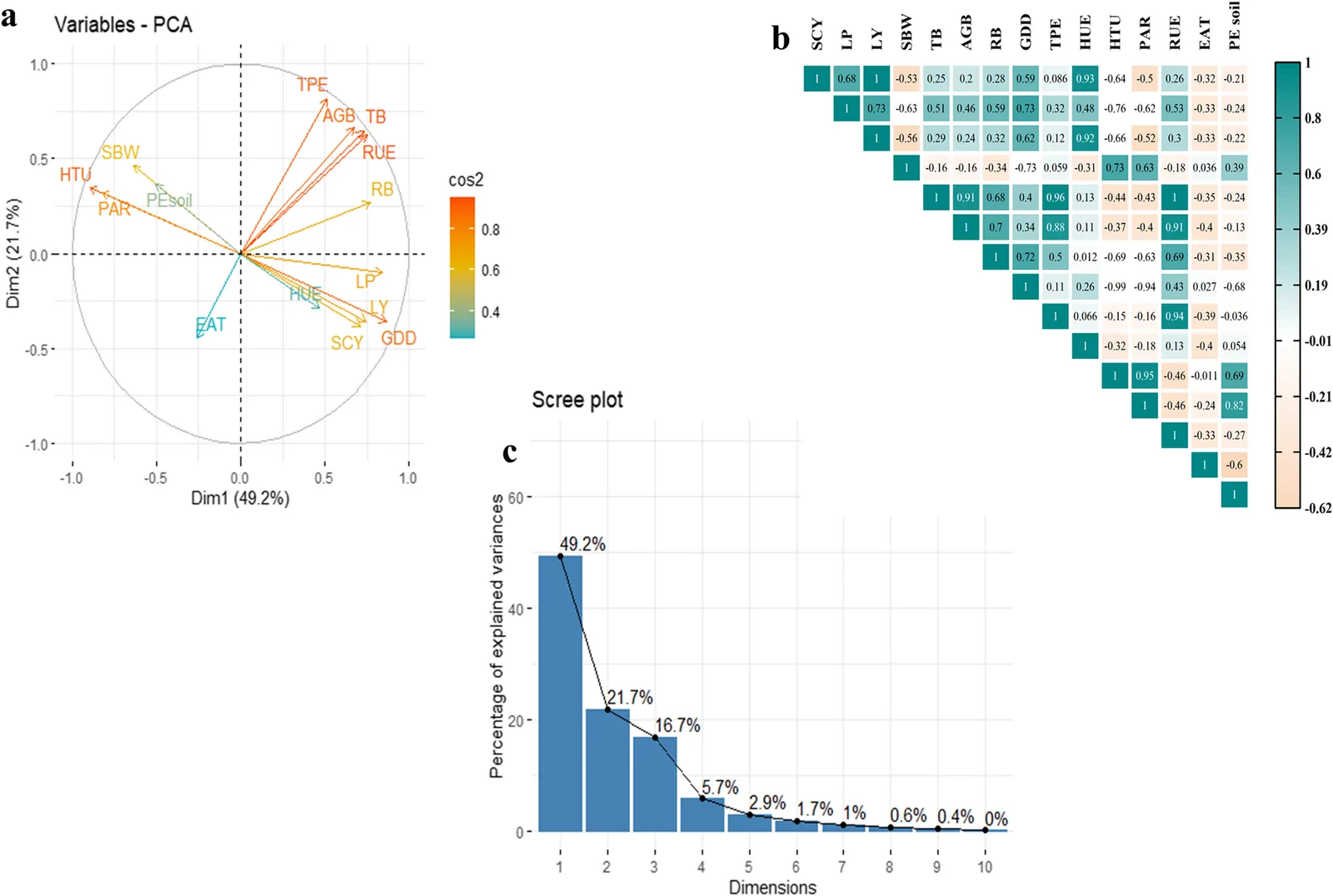
Relationship between yield, yield components, biomass, and resource utilization during cotton growth stage and development in three years. GDD, TPE, HUE, HTU, PAR, RUE, EAT, and PE soil represent the growing degree days, accumulated air temperature utilization efficiency, air heat use efficiency, heat unit index, photosynthetically active radiation, radiation use efficiency, effective accumulative temperature, soil heat production efficiency, respectively. SCY, LP, LY, and SBW stand for seed cotton yield, lint percentage, lint yield, and single boll weight, respectively. TB, AGB, and RB correspond to total, above, and reproductive biomass, respectively. **a** PCA, **b** Pearson correlation, and **c** Screen plot

In the PCA (top-left plot), 49.2% total variance is attributed to the first principal component (Dim1), and it is statistically significant considering that such variables as aboveground biomass (AGB), reproductive biomass (RB), and Accumulated air temperature use efficiency (TPE) contributed to it significantly, variables were standardized before PCA analysis. These variables also correlate strongly, positively, with seed cotton yield since they are all falling on the same side on the biplot (Fig. 7a). The second major factor (Dim2), and that is strong yet does not explain much of the variance, has less dominant but still relevant associations with other variables, such as EAT and accumulated PE soil, in the sense that it controls the seed cotton yield, though at shorter extent compared to biomass and thermal efficiency. This indicates that the seed cotton production depends on biomass, and thermal efficiency is the main motivator of seed cotton output in this dataset.

The scree plot (bottom-left plot) also validates the PCA results, indicating that the two key masses (first and second principal) did explain 70.9% of the total variance, with 49.2% of this variance explained by the first component. The sharp decline in the explained variance following the second dimension shows that the initial two dimensions explain the most significant trends in the information, indicating that biomass production and thermal efficiency are the most dominant variables to elucidate the change in seed cotton yield (Fig. 7c).

The correlation matrix (right plot) gives more information of relationships. There are significant positive relationships between Seed Cotton Yield (SCY) and AGB, RB, and TPE (0.68 to 0.91), which proves that an increase in biomass production and enhancing thermal efficiency is the key to cotton yields (Fig. 7b). SCY would also be significantly positively correlated with Growing Degree Days (GDD) (*r* = 0.91) and Heat Use Efficiency (HUE) (*r* = 0.72), which also supports the notion that favorable growing environments and efficient resource utilization play an important role in seed cotton yield. On the other hand, accumulated soil heat productive efficiency (PE soil) is negatively correlated with EAT (*r* = -0.42) and RUE (*r* = -0.68), indicating that when water consumption is excessive, the accumulation of temperature and use of radiation that contributes to the fullest utilization of cotton will be compromised. The matrix depicts that EAT is also weakly negatively correlated with some other variables, which illustrates the fact that excess soil water might inhibit other positive influences of the other growth factors.

### Comparing resource allocation and biomass production patterns across 2022-24

**Fig. 8:**
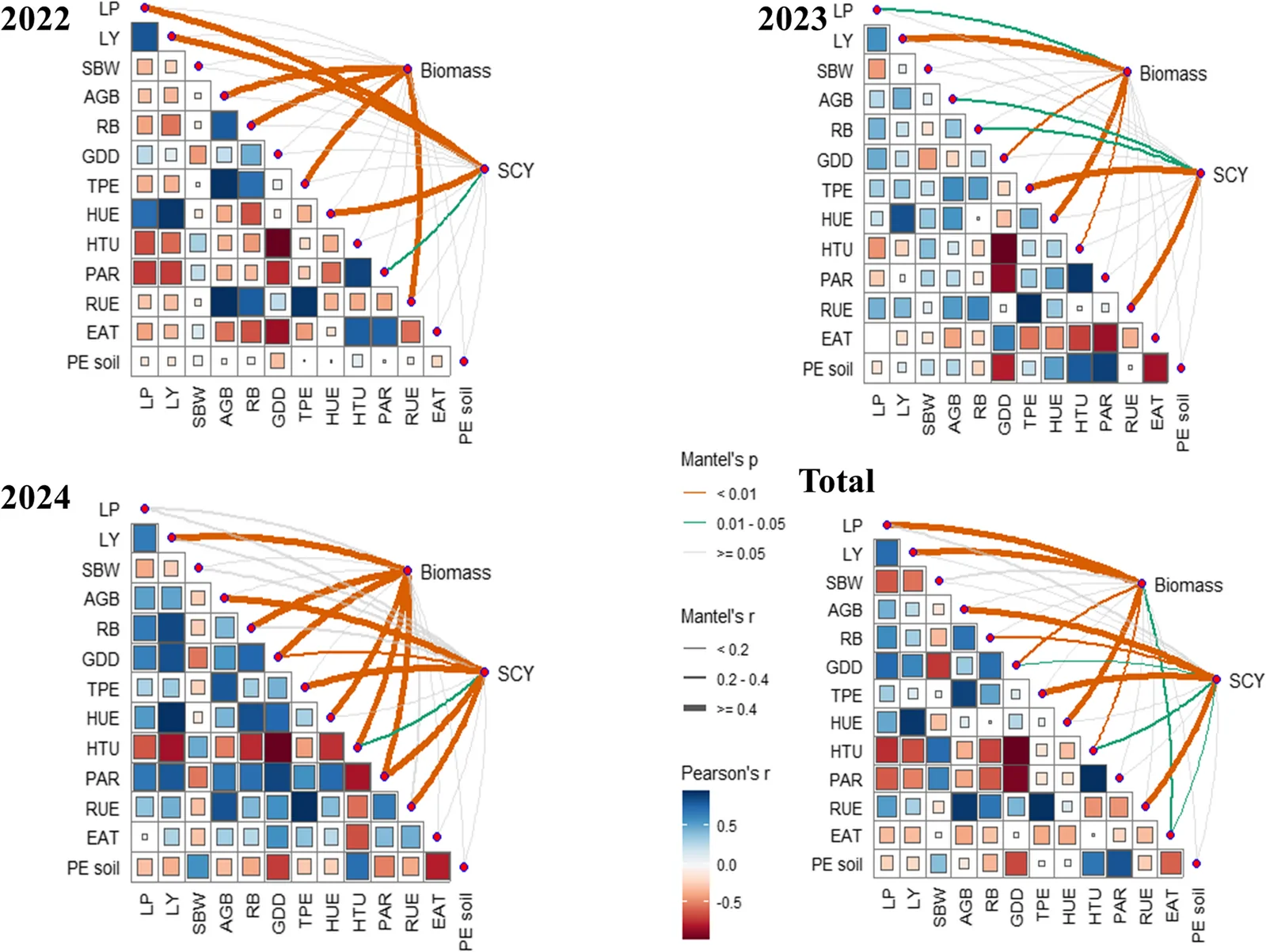
Pearson’s correlation coefficients and Mantel’s test relationships among different variables including lint percentage, lint yield (LP, LY), single boll weight (SBW), aboveground biomass (AGB), reproductive biomass (RB), growing degree days (GDD), Accumulated Air Temperature Efficiency (TPE), heat use efficiency (HUE), Heat Unit Index (HTU), photosynthetically active radiation (PAR), radiation use efficiency (RUE), Effective accumulative temperature (EAT), and accumulated soil heat productive efficiency (PE soil). Positive correlations are represented in blue, and negative correlations in red, with intensity indicating the strength of correlation. Orange connecting lines highlight significant Mantel’s correlations between biomass and seed cotton yield (SCY) across years (2022, 2023, 2024) and their combined total

Figure 8 shows the comparison of the patterns of correlation associated with the resource allocation of three years (2022, 2023, and 2024), and the final statement sums all three years together. The resource allocation to biomass production signs positive correlations prominently in consideration of key variables of growth, including LP, LY, SBW as well as AGB implying that resources are being effectively exploited to introduce biomass maximization (Fig. 8). These green arrows convey the high positive correlations of these growth parameters with the biomass, indicating that in 2022 the resources were efficiently distributed to maximize the growth and yield of the plant. However, other factors such as PAR and EAT show less strong correlations; the orange arrows represent a less significant contribution to the process of resource allocation that year.

The overall direction of resource distribution towards biomass is maintained in 2023, with a small shift. Although the data has a strong correlation between the biomass and growth variables, the strength of the correlations with the environmental factors, like TPE and HTU, is observed to decrease. This is supposed to imply that the allocation of resources will have altered in response to the dynamic environment conditions or a dissimilar management approach on the management of resource inputs in 2023 as compared to 2022 (Fig. 8). The R values of Mantel of these variables demonstrate that there is a change in the importance of some factors in the environment, and it may refer to possible alterations in the resources management approaches.

A stronger change in the distribution of resources is experienced by 2024. Correlations between biomass and the variables of growth such as LP, LY, and SBW are still positive, although the correlations with some of the environmental variables are reduced, indicated by an extended number of orange and gray arrows (Fig. 8). It means that the impact of some resources on biomass production may decrease in 2024, and the allocation strategy may have altered, or some environmental factors might have lost their role in biomass productions. For instance, variables like HTU, PAR, and TPE show weaker correlations with biomass, possibly due to resource scarcity, changing crop requirements, or altered environmental conditions in that year.

## Discussion

### The influence of climatic shifts on cotton production and resource management (2022–2024)

The three experimental years (2022–2024) showed clear climatic differences; however, the duration of the study is not sufficient to infer long-term climate trends. Therefore, the discussion focuses on how yearly variation affected soil temperature, water dynamics, and crop performance within this experiment (Fig. 1a). The strangest phenomena in 2022 were the intense variables in climatic conditions including the highest ever temperature increases, irregular changes between seasons as well as intermittent precipitation. Differences in weather conditions mainly influenced soil temperature accumulation and water consumption, which in turn affected biomass production and final yield. Although early sowing showed better model fitting for biomass accumulation, the highest yields were often obtained in later sowing dates, indicating that improved early growth efficiency did not always result in maximum yield. Differences in yield among sowing dates may be related to source–sink balance, where temperature conditions during vegetative growth influence assimilate production, while reproductive-stage temperature determines boll formation and retention. Nutrient availability also affects biomass accumulation and physiological activity, which may interact with temperature and water conditions [57].

Simultaneously, the relative humidity changed, which yielded acute shortages of precipitation and heavy rain. Such sharp swings are put in perspective in the wider context of human-induced climate change, where a study suggests that the presence of human activity has increased the vagaries of local weather formation [58, 59]. This instability must have brought up major challenges to crop production since unpredictable weather conditions for agricultural systems and made them less resource efficient [60, 61]. The 2022 seasonal changes in moisture and temperature raised a serious challenge to crop productivity, as crops are sensitive to humidity and temperature levels; shifts in farming regimes could result in inefficient resource distribution and sub-optimized crop performance and growth [62].

In 2023, the state of the environment started to stabilize as temperature swings decreased, and rain was observed in clearer predictable patterns. Precipitation in 2023 was less than in 2022, and there was also less magnitude of periodic changes in temperature. These trends refer to a variation in a more stable climate, which has been proposed in recent literature as a transition stage after decades of notable weather events [63, 64]. A more favorable crop-growing climate was encouraged due to decreased severity of extreme temperatures and precipitation events, as annual humidity became less volatile and moisture became more consistent across the year. This consistency in 2023 is a significant change towards predictability that could have a tremendous impact on the agricultural practices, as they could forecast the times of sowing and other seasons [42]. Besides, this predictable weather can decrease the risk of crop failures caused by unforeseen weather changes and increase total agricultural yields [65].

The greatest stabilization occurred in 2024 and is associated with the smallest interannual variability of meteorological conditions. This year was cooler than the previous year, with slight temperature variations, but not significantly different in humidity. However, there is a warming trend. Precipitation patterns in 2024 were relatively moderate with fewer extreme events, suggesting a broader move towards drier interglacial air masses. Consequently, such relative stability may be redefining the regional climate into a more predictable regime in a way that has always been linked with improved agricultural productivity [66].

### Effect of sowing dates on cotton growth and resource efficiency across years

The timing of sowing showed to be an important factor in cadencing cotton growth in order to exploit thermal capital to generate biomass. We found that the correspondence between EAT and biomass growth was improved by sowing at an earlier date, which reinforces the importance of an optimum sowing window to provide superior growth and yield. This relation follows the shape of logistic growth with an initial increase in biomass, followed by an increase in EAT to reach a plateau, the saturation level of plant growth potential. The accuracy of this relationship can be calculated by the coefficient of determination (R2), which fluctuates for the different sowing dates, reflecting a complex interaction involving thermal resources and plant development [28]. In years with an unusually high climate variability, e.g., 2022, the consistency of the logistic model in explaining the association between sowing dates and stock biomass accumulation was also inconsistent, indicating that the changing temperatures and irregular precipitation patterns of these years can confound the predictable relationship between EAT and biomass accumulation [16].

However, less variable weather conditions in later years, such as 2023 and 2024, resulted in higher model conformity with an R2 of over 0.95 on all sowing dates. This enhancement draws attention to the value of climatic conditions that are favorable to maximizing crop interactions and the use of resources [67]. Thermal resource capture was most favorable with early sowing dates, as these offered more time to vegetative growth, giving plants time to optimally balance the photosynthetic machinery and resulting in greater biomass production [68]. The effects of later sowing dates, whereby the time available to vegetative growth was negative, led to lower aboveground biomass accumulation, representing a partial inability to convert accumulated temperature into vegetative biomass.

Biomass accumulation, which includes aboveground and belowground biomass, also showed a positive relationship with early sowing dates. High values of R2 indicate a highly efficient use of thermal resources, so that the plants are efficiently transforming available energy to biomass. This efficiency is increased by early dates of sowing since this gives the crop ample growth time, thus increasing the total biomass accumulation [16]. Reproductive biomass, which is a decisive factor of cotton yield, was found to change according to sowing dates, especially in 2022. The strongest correlations between staging and thermal accumulation were in early sowing, with less conformity in late sowing dates. On the contrary, 2023 and 2024 were observed to have a better stability of reproductive biomass accumulation, providing high conformity across all the sowing dates; hence, supporting the advantage of stable weather patterns and optimization of reproductive growth.

### Impact of soil temperature on crop growth and thermal resource efficiency

Soil temperature forms an important determinant of plant growth because it determines both the biomass accumulation and the development of the vegetative structures, like the LAI. It is known that crop development and soil temperature, and more specifically, their effect on evapotranspiration, are strongly correlated. Root development is strongly regulated by environmental conditions, and deeper rooting improves the ability of plants to utilize soil water and nutrients [69]. Moreover, soil temperature has been noted as the major factor that affects root development and nutrient uptake, thus having a direct effect on the ability of a plant to utilize the photosynthetic process in the most optimal way possible [70, 71].

The interrelation between the sowing date and the soil temperature is of special concern, as the date of sowing influences the ability of a crop to utilize thermal resources optimally, and it subsequently relates to growth rates and the overall productivity [72]. Late sowing dates in particular are associated with higher soil temperature, especially in the upper layer, as the soil in the top layer gains time in building soil temperature throughout the growing season. This is congruent with findings that show that soils have a higher surface temperature of their soils to the environmental factors, such as sunlight and air temperatures, which bring more differences in temperatures with planting closer to the end of the season [73].

Furthermore, the thick layers of soil become less responsive to such temporal changes and heat up more slowly and gradually, which increases the risk of restricting the supply of thermal energy to roots at the initial stages of development [71]. This reduced responsiveness of deeper layers of soils to changing temperature highlights the necessity of understanding the behavior of different layers of soils to varying temperatures to implement best practices of crop management. Recently, it has been proven that adequate soil temperature has a potent influence on the physiological processes of plants. The soil temperature, especially during the initial crop growth, may boost root development and the efficiency of nutrient uptake, which subsequently contributes to the increased production of plant biomass and higher LAI [74]. Cotton root growth is optimal at soil temperatures of approximately 25–30 °C, while growth slows below 15 °C and declines above 35 °C, indicating that thermal conditions in the root zone strongly regulate crop development.

Nonetheless, high soil temperatures, especially in the upper levels, may impact the growth of plants unfavorably as well, which may accumulate in thermal stress and reduce the yield of crops [12]. Soil temperature effects were closely related to soil moisture, because water content influences soil heat capacity and root activity, thereby affecting crop growth. Consequently, the warmer the soil is, the higher the growth will be, but this has a limit, above which the positive effects become harmful. In addition, there is no linear relationship between soil temperature and the growth of crops. Efficiency in the use of thermal resources appears to increase with the growing season, especially when the soil temperature ceases to vary prematurely. Deeper soil layers showed more stable temperatures, which may have helped maintain root activity during later sowing dates when surface temperatures fluctuated. This aligns with recent studies indicating that the levels of soil temperature act as a driver of crop growth may strengthen over time, and the growth is more profound where thermal resources are better evenly distributed throughout the growing duration [41, 46]. In later sowing dates, higher surface soil temperatures may have accelerated early growth but could also increase stress during reproductive stages. Nevertheless, this effect of sowing date on thermal resources in the soil may not be constant each year, as it depends on annual changes in the climate, precipitation, and exposure to sunlight [46, 52, 75].

### Impact of soil depth on thermal accumulation and cotton growth patterns

However, the occupation pattern for 2023, characterized by weakening coordination with the environmental variables of TPE and HTU, suggests a shift in soil heat distribution. These developmental changes may simply be the result of changes in resource management practices or environmental changes. Comparable variation in the size of correlations over successive years of cotton production demonstrates the impact of changing environmental conditions and managerial practices on resource-allocation decisions and, hence, on subsequent effects on cotton yield. This agrees with previous research, which has determined that surface soils are quite vulnerable to climatic conditions, and they tend to warm more quickly than deeper green soils [74, 76]. The flattening response of EAT in 2024 implies that the thermal resource distribution at this shallow depth prevailed in an optimal state, indicating both an equilibrium between the soil temperature buildup and the thermal needs of the plant to grow. It has been proposed that during later stages of growth, and particularly closer to the surface level, thermal accumulation becomes stable, contributing to efficient seedling establishment and early vegetative growth [77–79].

On the contrary, the 30–70 cm depth showed a larger scattering of EAT values over the years, particularly in the year 2023, meaning that in the mid-soil layers, the dynamic thermal changes are greater. Such variability might be explained by the combination of multiple factors that include a variation in precipitation, cloud coverage, and the capacity of the soil to hold the heat [80]. Temperatures of the soil in this depth depend more on the thermal properties of the surface layer and the capacity of the underlying soil to store and dissipate heat. This will lead to a slower content of the mid-soil areas in a warming effect compared to the surface ones, and the variation that was experienced in 2023 could be relatively temporary climatic changes affecting resource distribution. Nevertheless, more intensive and consistent growth of EAT and, in 2024, implies that the thermal dynamics of the soil of this depth became adapted, which made its potential to retain heat in the past years, facilitating further plant growth at its reproductive stages [18].

The most gradual and slow rise in EAT took place at a 70–110 cm depth, which is in line with the properties of deep soil layers. Temperature variability at mid-depth (70–110 cm) suggests that this layer plays an important role in root-zone stability. The deeper soils are less exposed to solar radiation and, thus, warm more slowly compared to the surface and mid-soil [70]. This gradual thermal storage is frequently known as thermal inertia, and this is the desire that prevents a rapid increase in temperature in deeper soil layers. In this regard, the consistency of EAT in the depth studied over the three-year interval is an indication that the allocation of thermal resources was based on a rather conservative criterion of stability of thermal processes in the long run in preference to short-run time-dependent processes. Moreover, cotton roots can extend beyond 1 m depth, allowing plants to utilize water and nutrients from deeper soil layers. This observation conforms to the available literature that suggests that deeper strata of soil would act as thermal energy reservoirs that can sustain the growth of plant life in case temperatures in the surface soil become volatile [70, 71].

### Sowing timing and its effect on cotton yield, water, and radiation use efficiency

The earlier the sowing date (S1 and S2) in 2022, the lower the cotton yield was, yet the GDD values were greater than the later sowing date. The studies, including one by [81], have determined that early sowing dates can also result in sub-optimal crop-growth conditions because thermal accumulation and the water and radiation requirements of crops cannot be synchronized. These lower yields are probably due to the decreased efficiency of resource allocation at these early dates.

In 2023, there was a slight improvement in GDD at S1 and S2, relative to the year before, causing slightly higher yields, though the trend of high yield with later sowing dates was apparent. The highest yields this year (2023) were observed in sowing dates S5 and S6 because of the ideal fit between the thermal needs of the crop and other resources. The tendency was further enhanced by HUE at these later sowing dates, indicating that the crop had a greater capability of converting assimilates into yield because of more favorable environmental conditions. The same pattern was studied and found that a later sowing date allowed cotton to grow in favorable resource conditions and led to higher outputs [16, 42]. GDD showed the strongest correlation with yield because temperature accumulation directly controls crop development rate.

Ultimately, before 2024, all sowing dates improved significantly, with the most significant increase in the number of HUE, WUE, and RUE. This means that the timing details of the sowing to achieve appropriate conditions of thermostats, water, and radiation during the critical growth periods enabled the cotton plants to use the available resources efficiently, and the seed cotton yields were highest during the three years. Furthermore, excess water may reduce radiation use efficiency by limiting oxygen availability in the root zone. This trend was further complemented by the increase in the GDD values in 2024, implying that the crop would be able to capture more thermal energy when needed most and generate more biomass. The positive outcome of this enhancement on the capacity to allocate resources aligned with the results of a study by [41], who concluded that the utilization of radiation and heat could be optimized by means of the sowing dates, thus fostering the cotton output.

The linear trend of WUE was also similar across the years, where the later the sowing time, the higher the efficiency in using available water was exhibited. Lower values of WUE were observed during early sowing days (S1 and S2), which implies that water was not being properly used at the very critical stages of growth. Such inefficiency was probably because the early crop was subjected to low availability of water during its optimum growth periods. On the contrary, the superior WUE was demonstrated in later sowing dates (S5 and S6), which proved that these dates enabled the crop to make efficient use of water, indicating that regularity of sowing with the high-water availability increases the water use efficiency, which in turn increases the yield potential [42]. Higher WUE in later sowing may be related to improved synchronization between water supply and crop demand. Soil water balance strongly influences crop growth and irrigation efficiency, and proper scheduling is essential for improving water use under arid conditions [82]. Moreover, changes in soil water distribution can alter the regional water cycle, particularly in arid environments where soil moisture strongly controls crop performance [83]. Similarly, RUE across the study years (2022–2024) increased with the later sowing dates, with S5 and S6 achieving the highest RUE values. This means that the crop could trap and absorb amounts of solar radiation better at critical growth stages, which is paramount in boosting photosynthetic working energy and biomass accumulation [41]. Furthermore, excess water may reduce radiation use efficiency by limiting oxygen availability in the root zone.

### The role of water distribution in soil layers for optimizing cotton production

The negative coefficient of correlation between soil water use and seed cotton yield, which was relatively strong in the deeper soil profiles in 2022, confirms the adverse impact caused by unnecessarily high-water application in these profiles. This side effect of fertilizing demonstrates the importance of over-irrigation and irrigation-induced waterlogging, which can have a significant negative effect on cotton development through incomplete root oxygenation, leading to less nitrogen uptake and therefore limited potential yield [84–86]. Waterlogging, especially in deeper layers, inhibits soil aeration to cause suffocation of roots and their poor functionality, which may affect plant development directly [84, 87]. The findings of this year also indicated that, ineffectiveness of water distribution in deep layers leads to less cotton yield because the plant fails to reach the nutrients and oxygen, in case of soil oversaturation [8, 88]. This inefficient resource allocation, which results in excess water being drawn into deeper layers, translates into limitations on the levels of nutrients in the upper layers of soils, where the cotton plant’s active root system primarily resides [89].

Nevertheless, in 2023, there was an evident shift in the relationships between water consumption and yield. The decline of the negative correlation to deeper layers of the soil and the growth of the positive correlation to the middle level of soil in (x = 30–50, y = 40–70) show that the management of soil water has been adapted to better distribute water between the upper and bottom levels of the soil profile. Such alterations indicate that the cotton crop received more balanced water distribution, which is more important in enhancing cotton production, especially since the root system is likely to be concentrated towards the middle layers, where water is likely to be the most sensitive [52, 84, 89, 90]. This guarantees that the cotton plants can get the water that they require when they require it, especially during the reproductive phase of growth, which is essential when it comes to yield optimization. The relationship between deep soil water and yield suggests possible over-accumulation of water, although irrigation amounts were not measured directly.

The correlation between seed cotton yield and water consumption was most positive in 2024, with the upper and middle soil layers recording the highest positive correlation. The decrease in negative correlations in lower layers can indicate that the irrigation practices used this year were very productive and had access to the resources, and thus, there was an increase in cotton production. This also highlights the benefits of changing irrigation schedules depending on real-time screenings of the soil moisture, to maximize water availability, thus avoiding waterlogging and maintaining optimal cotton growth.

### Assessing the impact of biomass and environmental variables on cotton yield

Using the PCA analysis, the biomass-related variables, including AGB, RB, and TPE, have the greatest effect when determining seed cotton yield, with the first principal component (Dim1) contributing implicitly 49.2% to the variance. These results are in line with prior research, which highlights the importance of biomass under varied environmental conditions to enhance levels of crop production, specifically in cotton [91, 92]. PCA results confirmed that temperature accumulation and water distribution at different depths jointly influenced yield variation.

These positive correlations between seed cotton yield and these biomass-related measures (AGB, RB, TPE) are probable due to the corresponding evidence provided in the literature, which showed that improved biomass and thermal efficiency resulted in increased yields in cotton [41, 46]. Such findings support the claim that plant biomass is a key factor in crop productivity since it can support several physiological parameters that are crucial in crop development and yield. Moreover, recent studies have shown that the accumulation of biomass, particularly in relation to its above-ground components, is a useful predictor of seed cotton production in cotton cultivars [3, 93].

The environmental factors found to be relevant in describing seed cotton yield, but with lesser strength, are associated with the second leading component (Dim2), which shows relevance with EAT and PE soil. Although this relationship is not so dominant, it shows that temperature and water management are secondary, albeit significant, in the determination of cotton yields. Some studies have also indicated that water availability and accumulation of temperatures have a significant impact on cotton growth, with an optimal environment leading to a higher yield outcome [94, 95].

Nevertheless, the negative correlation of excess usage of soil water with EAT and RUE indicates that excess use of water can decrease other aspects of growth. This observation corresponds to the earlier studies that have indicated that when water is consumed beyond a certain threshold, it can impair the optimal use of solar radiation, and ultimately, reduce the growth potential of cotton [96]. Similarly, high soil moisture (especially at the flowering and the formation of bolls) conditions adversely affected the capacity of cotton to make use of solar radiation in photosynthesis [97].

Furthermore, the correlation further supports the significance of biomass production, as significant positive correlations were found between SCY and growth variables such as LP, LY, and SBW. This further supports the fact that effective distribution of resources with the aim of maximizing biomass is one of the main factors that define the cotton yield. This distribution of resources to above-ground biomass is essential in maximizing cotton yield, which was affirmed by the positive coefficient that exists between the biomass and yield factors [98].

Nonetheless, from the correlation patterns in 2023, we suggest a weakening of associations with indicators of environmental factors such as TPE and HTU, thus suggesting a changing strategy of allocating resources. Such developments may result from inconsistencies in resource management practices or, on the other hand, from modifications to the environment. Analogous variations in the strength of correlations in consecutive years in cotton production provided further evidence for the role of changing environmental and management practices in the allocation of resources and for consequent impacts on cotton yield [42, 94, 99]. Furthermore, improving crop management under changing climate conditions is essential for enhancing agricultural resilience [100, 101].

## Conclusion

The current study highlights the importance of sub-optimizing sowing dates to promote cotton productivity and resource-use efficiency in the heterogeneous climatic environment. The results suggest that the optimal sowing window under the conditions of this study was approximately early to mid-May. Indirect evidence shows that the growth of cotton, biomass, and yield strongly depend on the timing of their sowing since this factor regulates the distribution of the necessary resources. Treatments S5 and S6 with a later sowing schedule (when sowing fits a stronger thermal accumulation curve) showed higher quality crop production and thus made better use of resources. On the contrary, the result of the treatment S1 and S2 in the form of earlier sowing was linked with lower outputs due to a lack of mobilization of resources at the key vegetative and reproductive stages. The above study additionally outlines the most common climatic parameters, such as the GDD, HUE, the WUE, and RUE. Also, in the analysis, the effect of soil temperature gradients within 0–30, 30–70, and 70–110 cm depths on resource allocation and plant development is explained. The previous sowing dates had an undesirable impact on heat-storage capacity that was related to a deeper soil stratum, thus influencing cotton productivity negatively. Given the increasing climatic variability due to anthropogenic global change, fine-tuning of sowing dates can be a feasible option when it comes to controlling cotton growth in accordance with the favorable meteorological conditions. Moreover, limitation of this study is that it was conducted over only three growing seasons. Whereas the results can be applied practically to the management of cotton in the future; in particular, the timely sowing of cotton can lead to better performance, where more resources are utilized wisely and the harmful effects of weather conditions and severe weather phenomena are avoided. Understanding soil temperature dynamics at different depths provides a useful basis for optimizing sowing date and improving cotton productivity under variable climatic conditions.

## Data Availability

Data will be made available on request.
